# Disinfection Strategies for Poly(methyl methacrylate): Method Sequence, Solution Concentration, and Intraoral Temperature on Antimicrobial Activity

**DOI:** 10.3390/polym17010008

**Published:** 2024-12-24

**Authors:** Ana Beatriz Sato Kamio, Andressa da Silva Barboza, Maria Eduarda Broering da Silva, Artur Ferronato Soto, Juliana Silva Ribeiro de Andrade, Thais Mageste Duque, Ariadne Cristiane Cabral da Cruz, Ricardo Ruiz Mazzon, Maurício Malheiros Badaró

**Affiliations:** 1 Department of Dentistry, Federal University of Santa Catarina (UFSC), Av. Delsino Conti, s/n—Trindade, Florianópolis 88040-900, SC, Brazil; anabeatrizsatokamio@gmail.com (A.B.S.K.); andressahb@hotmail.com (A.d.S.B.); mariaeduardabroering@outlook.com (M.E.B.d.S.); arturfsoto@gmail.com (A.F.S.); sribeirooj@gmail.com (J.S.R.d.A.); thaismadu@gmail.com (T.M.D.); ariadne.cruz@ufsc.br (A.C.C.d.C.); 2 Department of Microbiology, Immunology and Parasitology, Federal University of Santa Catarina (UFSC), Av. Delfino Conti, s/n—Trindade, Florianópolis 88040-900, SC, Brazil; ricardo.mazzon@ufsc.br

**Keywords:** *Candida*, acrylic resins, dentures, disinfection, temperature

## Abstract

This study aimed to evaluate the antimicrobial effectiveness of different disinfection protocols for dentures by combining methods, varying intervention sequences, sodium hypochlorite (NaOCl) concentrations (0.1% and 0.25%), and post-exposure to intraoral temperature. The heat-polymerized poly(methylmethacrylate) (PMMA) was divided into groups (n = 15): control (C, distilled water immersion), B (brushing), I0.1% and I0.25% (isolated NaOCl immersion), B + I0.1% and B + I0.25% (brushing followed by immersion), I + B0.1% and I + B0.25% (immersion followed by brushing), and B + I0.1% + T and B + I0.25% + T (brushing, NaOCl immersion, and overnight exposure to 35 °C ± 2 °C). The post-disinfection exposure to intraoral temperature simulated the denture use during sleeping time. Quantitative evaluation was performed by colony-forming unit (CFU/mL) counting of *C. albicans* and qualitative analysis by scanning electron microscopy (SEM) images. Data were processed by one-way ANOVA with Tukey’s post-hoc test to compare different protocols at the same concentration and among groups (α ≤ 0.05). Applying 0.25% NaOCl in associated protocols, the intervention sequence was no different (B + I and I + B) and caused the lowest *C. albicans* counts. The 0.1% NaOCl lost part of its action when the immersion method started the protocols. B + I0.25%, I0.25% + B, and B + I0.1% had similar antimicrobial efficacy, but the intraoral temperature (B + I + T) reduced the efficacy of these protocols, regardless of NaOCl concentration. Residual biofilm recolonization was also detected in SEM images. In conclusion, all the combinations between mechanical and chemical methods using 0.25% NaOCl were the most effective against *C. albicans*. The antimicrobial efficacy of NaOCl at 0.1% changes depending on the intervention sequence. The intraoral temperature influenced the *C. albicans* recolonization after the disinfection protocols.

## 1. Introduction

Worldwide forecasts indicate an increase in the need for total or partial prostheses, as the world population aged over 65 is expected to double by 2050 [1]. Complete prosthetic rehabilitation in these individuals involves restoring masticatory function, and aesthetics, consequently improving the quality of life [2]. Heat-polymerized poly(methylmethacrylate) (PMMA) is widely chosen as the base material for prostheses due to its ease of manipulation, cost-effectiveness, favorable aesthetic properties, and relatively low toxicity [3]. However, the incorporation of air and the presence of free monomers even after the complete polymerization of the material result in a surface with porosities, that provide a suitable niche for the formation and retention of biofilm, causing infections in oral and systemic mucosa [4].

Prosthesis-related stomatitis (PRS) clinically manifests as a chronic erythematous inflammation of the prosthesis-covering region [4,5,6]. It is the most common lesion of the oral mucosa associated with denture users [7], affecting approximately 35 to 50% of individuals, with a multifactorial etiology and various risk factors, including poor hygiene, continuous nighttime prosthesis use, and misfit [8]. Additionally, systemic factors such as immunosuppression and a history of smoking [8] can exacerbate the severity of the disease in patients. The primary microorganism found in total prostheses of PRS-affected patients belongs to the *Candida genus* [6,9]. *C. albicans* is the most isolated pathogen, acting as an opportunistic microorganism in the form of hyphae or yeast [4,10]. Formation of biofilm capacity is a *C. albicans* pathogenesis [10]. The *C. albicans* biofilms represent a significant clinical challenge as they are inherently resistant to conventional antifungal interventions, host immune response, and environmental factors [10,11,12].

The biofilm identified on the surface of prostheses is characterized as complex and synergistic, with an intricate network of various bacterial and fungal species embedded mainly in crack and irregularity regions, and with a protective extracellular matrix layer [13]. Thus, prosthetic devices can become a reservoir for potentially pathogenic microorganisms [14], influencing the onset or aggravation of gastrointestinal infections, fungal endocarditis, and respiratory problems, especially in older and/or immunocompromised patients [10,15,16]. This biofilm accumulation is exacerbated when combined with continuous prosthesis use overnight [13].

To prevent PRS, a disinfection routine is essential [5,6]. When it comes to biofilm removal from the prosthesis, a combination of mechanical and chemical methods is recommended [6,7,14], followed by prosthesis removal during the nighttime [6]. Sleeping with the prosthesis is associated with a higher risk of developing PRS, as it reduces the protective effect of saliva, tongue cleaning action, and good mucosal oxygenation [9]. Brushing (mechanical method) is a simple, efficient, and affordable option. However, previous studies show that the isolated use of this method is not sufficient to reduce fungal and bacterial colonization on the PMMA surface [17]. Moreover, brushing can be challenging for older patients with motor limitations, leading to inadequate prosthesis hygiene. Therefore, additional interventions are needed for better microbial control. In addition, the use of disinfectant immersion solutions is necessary, as the rough topography of the prosthetic surface increases biofilm retention and accumulation, making it difficult to remove with isolated methods [17,18,19,20].

In the literature, sodium hypochlorite (NaOCl) is regarded as the reference standard [6,18,19]. NaOCl directly dissolves the polymeric structure of the organic matrix of accumulated biofilm on the prosthesis. The 0.25% NaOCl concentration has shown effectiveness in removing biofilm from the prosthesis [6,14] and indirectly controlling mucosal colonization in individuals with PRS [14]. Concentrations of 0.1% have also succeeded in decreasing biofilm on prostheses and achieving better results in PRS remission [14]. However, it is important to conduct antimicrobial research involving several clinical disinfection protocols to indicate the most effective sequence of methods. Additionally, it is necessary to establish the ideal NaOCl concentration within these protocols. Lastly, it is crucial to evaluate the influence of exposure to intraoral temperature after disinfection, as this would reproduce the individuals who do not remove their dentures at night. Therefore, this study aimed to define the most antimicrobial-effective cleaning protocol against *C. albicans* biofilm on PMMA, varying the sequence of methods (mechanical and chemical), NaOCl concentration (0.25% and 0.1%), and post-disinfection exposure to intraoral temperature. The null hypothesis would be that there was no difference between the protocols using a combination of methods.

## 2. Materials and Methods

### 2.1. Study Design and Sample Size Calculation

This in vitro study had antimicrobial effectiveness as the response variable. The varying factors include varied sequences of intervention, different concentrations of NaOCl, and exposure to intraoral temperature after the most effective protocols. Quantitative analysis was performed through colony-forming units (CFU/mL) counting, and qualitative analysis involved scanning electron microscopy (SEM) photomicrographs. The sample size calculation was based on the study by Badaró et al. [19] for the evaluation of microbial load for *Candida albicans*. Using GPower© 3.1.9.4 (Heinrich-Heine-Universität, Düsseldorf, Germany), information regarding the estimated effect size (f = 0.40—large effect size), alpha error probability of 5% (*p* < 0.05), and test power of 0.80, considering the one-way ANOVA test with a maximum of 10 groups, the required sample size was 165 samples, with 15 in each group.

### 2.2. Specimen Fabrication and Group Formation

The heat-polymerized PMMA (Classico; Artigos Odontologicos Classico Ltda, Sao Paulo, SP, Brazil) was manipulated by mixing the resin (powder) containing polymethylmethacrylate, benzoyl peroxide, and biocompatible pigments with the liquid containing methylmethacrylate monomer and inhibitor. Rectangular samples of heat-polymerized PMMA were obtained from molds made with metal matrices (90 × 30 × 4 mm). Metal matrices were included in dense condensation silicone (Zetalabor, Zhermack S.p. A, Badia Polesine, Rovigo, Italy), which was embedded in number 07 flasks (OGP Produtos Odontológicos Ltda, Sao Paulo, SP, Brazil), using type III gypsum (Gesso Rio, Orlando Antônio Bussioli ME, Rio Claro, SP, Brazil). The heat-polymerized PMMA was pressed at 1250 Kgf for 30 min in the molds, according to the manufacturer’s instructions, and polymerized in a water bath [14].

Two different rough surfaces form complete dentures, the external one with an extensive polish and the internal surface without any polish procedure, which is maintained in contact with the palate mucosal. To simulate both characteristics of a complete denture’s roughness, the surfaces of the specimens were finished and standardized by a roughness tester (SJ201P Surftest; Mitutoyo Corporation, Kawasaki, Kanagawa, Japan). The internal surface simulated had the rough values standardized at 2.7 to 3.7 µm [21]. Tungsten carbide Minicut^®^ burs (American Burrs^®^, Palhoça, SC, Brazil), brushes of different grits (fine, medium, and coarse), and 120-grit sandpaper (Norton Industria Brasileira, Guarulhos, SP, Brazil) were used [14]. The external surface simulated had rough values standardized in values under 0.2 µm, which is the limit to avoid microbial adhesion [22]. It was polished using a horizontal polisher (DP 9; Ballerup, Copenhagen, Denmark) with 150-, 220-, 400-, 600-, 1200-, and 2000-grit abrasive papers (Norton Industria Brasileira, Guarulhos, SP, Brazil) and a wet rag wheel with calcium carbonate (Orlando Antonio Bussioli ME, Rio Claro, SP, Brazil) [23]. To fit the specimens into the wells of 48-well culture plates, the PMMA specimen was cut using carborundum discs (Dentorium Products Inc.; Farmingdale, NY, USA) mounted on a mandrel (Microdont, São Paulo, SP, Brazil) and a handpiece and contra-angle handpiece (Smartmatic S10, Kavo do Brasil Indústria e Comércio Ltda., Joinville, SC, Brazil), into square shapes with dimensions of 5 × 5 × 2 mm, individually verified with a digital caliper (CD6 CSX-B, Mitutoyo Sul Americana Ltda, Sao Paulo, SP, Brazil). The non-polished side was used for biofilm formation to simulate the internal surface of complete dentures which remains in contact with the palate mucosa and is colonized by Bacteria and *Candida* spp. [6,23]. A lateral marking on each specimen indicated the unpolished surface.

Subsequently, all cut specimens were sterilized by ethylene oxide gas diffusion [24] (ISO 11135 [25]). Sterilization was programmed in the 1st cycle with a 20 min pre-vacuum at 55 °C, 180 min of sterilization, 120 min of hyperventilation, a pressure of 50 kgf/cm^2^, and a vacuum of 50 kgf/cm^2^.

### 2.3. Hygiene Protocols and Group Formation

**Figure 1:**
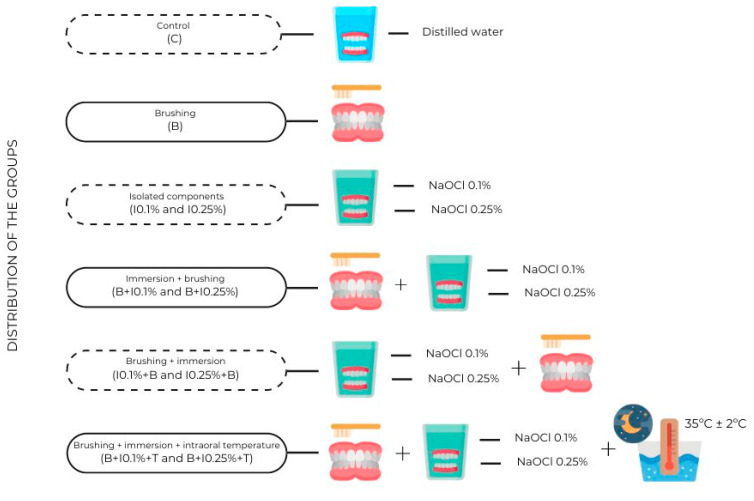
Distribution of control and experimental groups according to disinfection protocols. Created with Canva.com.

The chosen microorganism for the tests was *C. albicans* (ATCC 10231). The disinfection protocols, the concentration of NaOCl used, and the exposure to intraoral temperature determined the distribution of the specimens in the groups (Figure 1). Isolated protocols (brushing or immersion), combined protocols (brushing followed by immersion or immersion followed by brushing), and exposure to intraoral temperature after the combined protocols most efficiently were applied. To ensure the sterile condition of the specimens, a negative control group was immersed in a 48-well plate with 1 mL of Sabouraud Dextrose Broth without inoculation of the fungal species. The positive control involved inoculating the specimen with *C. albicans* in Sabouraud Dextrose Broth.

Each group aimed to simulate patient-established disinfection protocols for complete dentures based on the dentist’s recommendations [6,23]. Mechanical and chemical methods were simulated. A single operator, previously calibrated, used a standardized brushing movement on both sides of the specimen. A soft toothbrush was applied to it (K’ Dental Comércio de Materiais Odontológicos), immersed in a solution of 20 mL distilled water and 5 mL neutral liquid soap (Beira Alta Cosméticos Ltd.a, Guarulhos, SP, Brazil). The brushing time was defined according to Ramage et al. [26], in which the 2 s was calculated based on the proportional surface area of an upper denture to be brushed for an average of 2 min.

The chemical method involved immersion in NaOCl at concentrations of 0.1% and 0.25% (Perfecta Farmácia de Manipulação Ltda, Florianópolis, SC, Brazil) for 20 min [6,7,14,19,23]. The NaOCl concentrations were obtained in partnership with a compounding pharmacy. To ensure the solution was at the correct concentration, it was used on the same day as its manipulation. The specimens were fully submerged in microtubes (Kasvi Importação e Distribuição de Produtos Para Laboratórios Ltda, São José dos Pinhais, PR, Brazil) with 1 mL of the NaOCl liquid solution. To simulate the use of dentures after disinfection by the patient at night, the specimens were immersed in distilled water for 8 h in microtubes (group: B + I + T) and placed in a microbiological oven at 35 °C ± 2 °C. The intraoral temperature was following Kameyama et al. [27]. This procedure was added to the combination protocols following the implementation of the brushing and chemical disinfection method. Thus, seeding procedures were only carried out after the 8 h overnight period. The artificial saliva was not used, due to its influence on *C. albicans* growth [28,29]. Groups not subjected to any type of disinfection were placed 3 times in saline solution.

### 2.4. Main Variables

#### 2.4.1. Microbial Growth

To form the monospecies biofilm, *Candida* strains were thawed and reactivated in Sabouraud Dextrose Broth (Kasvi Importação e Distribuição de Produtos para Laboratórios Ltda, Sao Jose dos Pinhais, PR, Brazil). They were stored in a bacteriological incubator (Incubator shaker Banho Dubnoff NT232, Nova Técnica Ltda, Indústria e Comércio de Equipamentos para Laboratório Ltda, Piracicaba, SP, Brazil) at 37 °C for 24 h. The *C. albicans* inoculum was standardized by microbial counting based on McFarland Scale 0.5. It was then adjusted to a concentration of 1 × 10^6^ cells/mL. The wavelength used was 625 nm, with an absorbance value between 0.085 and 0.141. To confirm standardization, a complementary count was carried out in a Neubauer chamber (HBG, Alemanha). For correct standardization, 0.1 mL of the inoculum was diluted to 9.9 mL of Sabouraud Dextrose Broth [30].

#### 2.4.2. Microbial Inoculation and Biofilm Formation

With the sterile specimens, each one was individually placed in a well of a 48-well culture plate (Kasvi Importação e Distribuição de Produtos Para Laboratórios Ltda, Sao Jose dos Pinhais, PR, Brazil) within the laminar flow hood. The distribution ensured that each specimen’s rough surface faced upward, promoting biofilm adherence. Using a sterile pipette tip, 1 mL of culture medium containing the standardized *C. albicans* inoculum was pipetted onto each specimen, except for the negative control group, which received sterile culture medium. To ensure *C. albicans* adherence to the specimens, the 48-well plates were placed under agitation (75 rpm) for 1 h and 30 min in a microbiological incubator at 37 °C [24]. The agitation procedure used an OS-20PRO circular shaker (Joanlab Equipment CO., LTD., Huzhou City, Zhejiang Province, China), allowing the culture plates to be held by rods for greater movement control.

After this time, the entire contaminated medium in each well was removed for washing with sterile 0.9% saline solution (NaCl) to eliminate non-adhered microorganisms [24]. Subsequently, each well was filled again with 1 mL of sterile culture medium and incubated at 37 °C under agitation (75 rpm). After 24 h, to avoid medium saturation, half of the medium was replaced with fresh culture medium. The biofilm growth was completed after 48 h of incubation. Thus, the specimen and biofilm assembly were ready for the implementation of the disinfection protocols for each group. After completing the disinfection protocols, the groups not subjected to distilled water immersion were placed in microtubes containing saline to remove soap residues. Then, each specimen was immersed in a test tube containing saline and subjected to a Vortex mixer (Vortex Mixer VM 370, Intllab, Seri Kembangan, Selangor, Malaysia) for 1 min.

**Figure 2:**
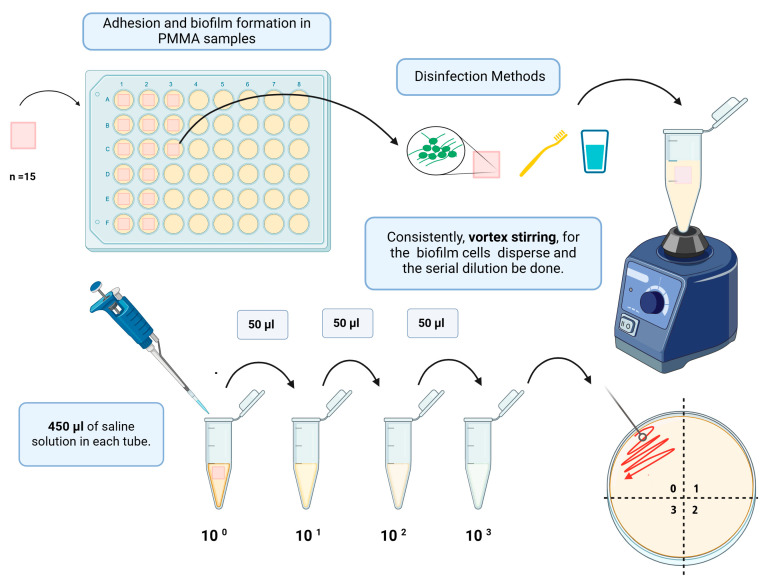
Serial dilution was performed in microtubes for seeding Petri dishes. Created with BioRender.com.

Subsequently, a 50 µL aliquot of the test solution was directly seeded (pure sample, 10^0^) onto a Petri dish (Petri Dish FL3-9015RI, FirstLab, São José dos Pinhais, PR, Brazil) containing Sabouraud Dextrose Agar (Biolog Brasil Distribuidora Ltda, Sao Paulo, SP, Brazil) (Figure 2). Serial dilutions were then made [1:10 (10^−1^); 1:100 (10^−2^); 1:1000 (10^−3^)]. Each dilution was transferred to the agar culture medium (0.025 mL) in the respective quadrants, incubating for 48 h at 37 °C. The CFU/mL calculation considered the dilution where the number of colonies ranged from 0 to 300, with “ᵑ” being the absolute value of the dilution considered (0, 1, 2, and 3), and “q” being the quantity (mL) pipetted for each dilution of the seeding [30]. The formula used was CFU/mL = number of colonies × 10ᵑ/q.

#### 2.4.3. Scanning Electron Microscopy (SEM)

After the disinfection protocols were completed, samples from the experimental and control groups were qualitatively analyzed through scanning electron microscopy (SEM), obtaining high-resolution images with depth of field for magnifications up to 10,000×. The samples were stabilized by chemical fixation with 2.5% glutaraldehyde, washed in a buffer solution to remove excesses, and dehydrated [20].

Dehydration was carried out slowly and gradually through immersions in increasing ethanol concentrations from 30% to 100%. Once reaching the final maximum concentration, the specimens were dried in a critical point apparatus and transferred to the stub for metallization. For metallization, the specimens were fixed on an aluminum base to undergo the sputtering process for 2 min. Gold was chosen as the conductive layer for its high efficiency in depositing irregular objects. In this way, images at magnifications of 10,000× were obtained.

### 2.5. Statistical Analysis

Data analysis was conducted using statistical software programs, including Sigma Plot 12.0 by Systat Software Inc. (Point Richmond, CA, USA) for statistical analyses and Graph Pad Prism version 10.0.0 (Boston, MA, USA) for graphical representations. The data underwent assessment to confirm normal distribution and homogeneity of variance. The *t*-test for independent samples, considering Brown–Forsythe’s test for equality of variances, was used to compare concentrations. Dependent variables were subjected to a 1-way analysis of variance (ANOVA), followed by the Tukey significant difference post hoc test (*p* < 0.05) and Dunnett’s multiple comparisons test to determine significant differences among groups.

## 3. Results

### 3.1. Colony-Forming Units per Milliliter (CFU/mL)

**Figure 3:**
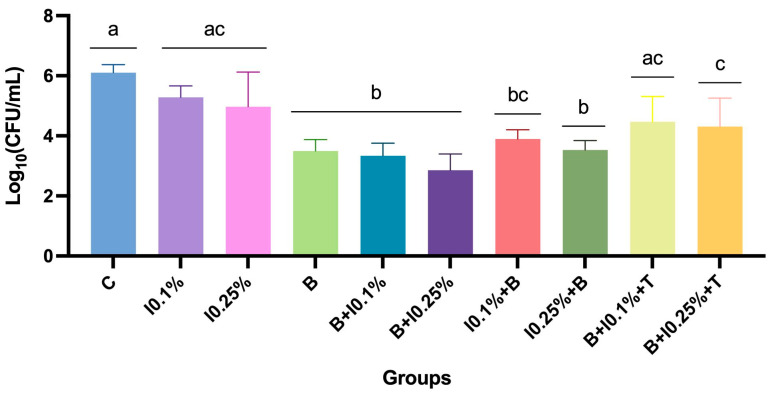
Mean ± standard deviation of log-transformed colony-forming units per milliliter [log_10_(CFU/mL)] for all protocols and NaOCl concentrations. Different letters indicate significant differences among groups (*p* < 0.05).

The analysis of the antimicrobial efficacy of disinfection protocols revealed significant differences among the treatments (Supplementary Table S1). Combining brushing with neutral soap followed by immersion in 0.25% NaOCl demonstrated the highest microbial control compared to other methods. B + I0.25% promoted a reduction of three log10 compared to the control group (Supplementary Table S2). Conversely, the control group and methods employing NaOCl immersion alone exhibited the highest mean colony-forming units per milliliter (CFU/mL) counts (*p* < 0.0001; Figure 3). Protocols incorporating a combination of methods and overnight exposure to intraoral temperature presented intermediate results, with an increase in microbial counts. Notably, the sequence of interventions promoted a difference between the protocols using NaOCl at 0.1%, in which brushing before immersion was preferred.

**Figure 4:**
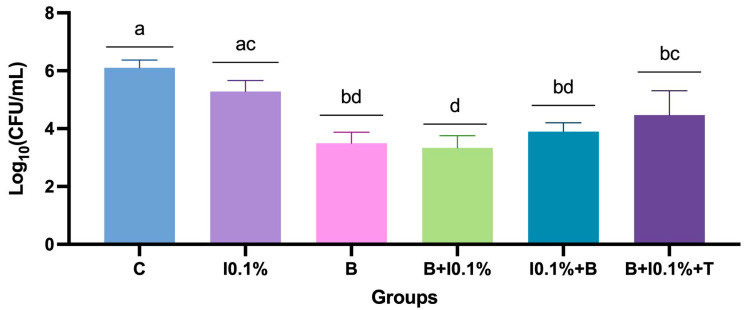
Mean ± standard deviation of log-transformed colony-forming units per milliliter [log_10_(CFU/mL)] for the comparison of groups based on NaOCl concentration of 0.1%. Different letters indicate significant differences among groups (*p* < 0.05).

Considering only the NaOCl at 0.1% related protocols, it was evident that groups B, B + 0.1%, and I0.1% + B exhibited significant differences among them, emerging as the most effective antimicrobial protocols. The combination of methods followed by exposure to intraoral temperature (B + I0.1% + T) presented intermediate results, showing a notable increase in microbial counts. The 0.1% NaOCl immersion (I0.1%) alone and control groups obtained the least favorable results (*p* < 0.0001) (Figure 4).

**Figure 5:**
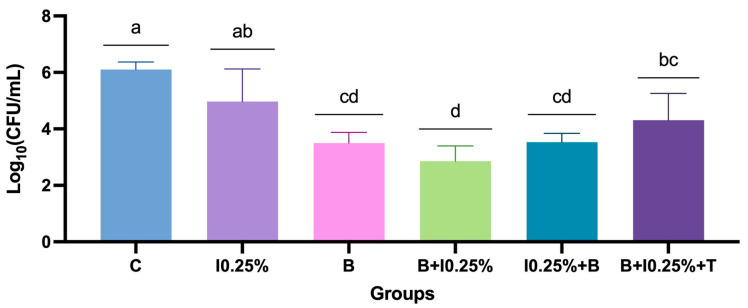
Mean ± standard deviation of log-transformed colony-forming units per milliliter [log_10_(CFU/mL)] for comparing groups based on NaOCl concentration of 0.25%. Different letters indicate significant differences among groups (*p* < 0.05).

The examination of protocols related to NaOCl at 0.25% revealed that the protocol combining brushing with immersion yielded superior antimicrobial efficacy. Isolated brushing (B) and brushing combined with NaOCl (B + I0.25%; I0.25% + B) and with controlled temperature (B + I0.25% + T) showed similar values (*p* > 0.05), unlike the control group and only immersion in NaOCl (I0.25%) (*p* < 0.05) (Figure 5).

Significant differences were observed when comparing protocols with the same intervention at varying sodium hypochlorite (NaOCl) concentrations, specifically between the groups employing brushing followed by immersion and those featuring immersion followed by brushing (*p* = 0.002) (Table 1).

### 3.2. SEM Analysis

**Figure 6:**
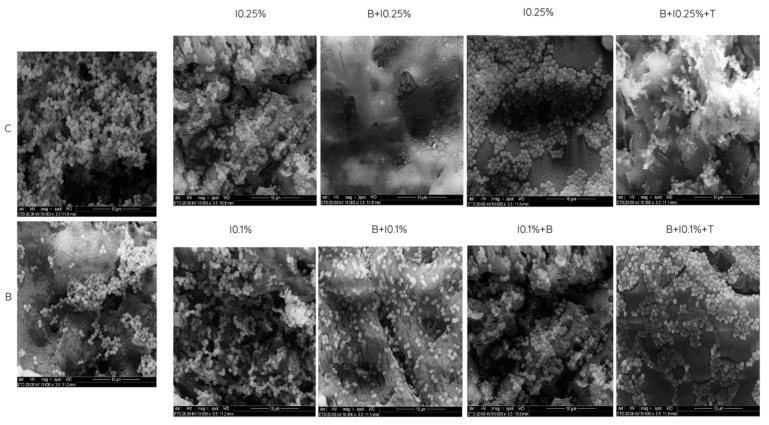
Scanning electron microscopy (SEM) images of the representative PMMA surface demonstrating the presence of *C. albicans*.

In the qualitative analysis based on scanning electron microscopy (SEM) images, fungal cell penetration into the poly(methyl methacrylate) surface, especially in porous regions, was observed (Figure 6). The morphology of *C. albicans* was predominantly yeast-like, the most common form when seeded individually [31]. The visualized biofilm accumulation aligned with the CFU/mL count results. Images showed a lower biofilm accumulation in the B + I0.25% group compared to the control group and other protocols. Conversely, groups with isolated immersion, regardless of NaOCl concentrations, exhibited a significant biofilm accumulation on the surface. Protocols combining methods followed by overnight exposure to intraoral temperature displayed an intermediate biofilm presence between the control group and the most effective protocol (B + I0.25%).

## 4. Discussion

A denture base made of acrylic resin comprises polymers of acrylic acid, methacrylic acid, or acrylonitrile [32]. It can also refer to a group of thermoplastic resins made by polymerizing esters of acrylic or methylmethacrylate acids [32]. Acrylic resin, also known as poly(methyl methacrylate), has a Knoop hardness index ranging from 18 to 20, a tensile strength of approximately 60 MPa, a density of 1.19, and a modulus of elasticity of around 2.4 GPa [32]. Conventional full dentures are typically made using heat-cured acrylic resin. They can also be manufactured using CAD-CAM milling and 3D printing [33]. Regardless of the material used, it is crucial to maintain effective hygiene protocols to control microbial biofilm, which is essential for both local and systemic health. Thus, the results of this study indicate differences in antimicrobial effectiveness among disinfection protocols, rejecting the null hypothesis.

The associated protocol with the sequence of brushing before chemical immersion in NaOCl at 0.25% (B + I0.25%) without post-exposure to intraoral temperature caused the highest reduction in *Candida* counts (CFU/mL). According to ISO 14729 [34], this requires a 99.9% reduction in bacteria and a 90% reduction in yeasts and molds [35]. The B + I0.25% protocol meets the criteria for reducing *C. albicans*. In contrast, when this protocol was associated with post-exposure to intraoral temperature, there was a recolonization by *C. albicans.* In addition, independent of NaOCl concentration, the isolated chemical immersion protocols (NaOCl0.1% and NaOCl0.25%) had the lowest reductions in fungal load compared to the control group. Thus, corroborating the literature, it was reaffirmed that the combination of mechanical (brushing) and chemical (immersion) methods is the most suitable protocol for microbial control of complete dentures [6,17,36]. In complement, this research addresses the knowledge gap related to the significance of appropriate disinfection protocol strategies. The sequence of methods (B + I or I + B), the concentration of NaOCl (0.1% or 0.25%), and the influence of intraoral temperature after disinfection protocol (overnight, 8 h) were found to cause differences in the microbial behavior of *C. albicans*.

This study proves that the sequence of disinfection methods and NaOCl concentration influenced the antimicrobial effectiveness of the protocols. The most effective concentration of NaOCl was 0.25%, which obtained the lowest microbial load, regardless of the sequence of methods (B + I0.25% and I0.25% + B) being similar. Both protocols, without being post-exposed to the oral temperature, guaranteed a reduction of at least two log10 when compared to the control group. According to ISO 14729, these found are acceptable [35]. Clinical trials using the same NaOCl concentration (0.25%) and varying the sequence between both methods, mechanical and chemical, indicated superior efficacy than the other protocols analyzed [6,14,37]. Based on this, it can be assumed that 0.25% NaOCl is a sufficient concentration to maintain the anti-candidal action, regardless of the sequence of methods. In contrast, the concentration of 0.1% NaOCl showed a difference in effectiveness according to the sequence of disinfection methods. It is supposed that the NaOCl concentration has decreased to a point, where its antimicrobial potential was considerably reduced. Thus, the group that combined brushing followed by immersion in 0.1% NaOCl (B + I0.1%) was similar to brushing alone, while I0.1% + B obtained major microbial growth. This suggests that the ability to reduce the *C. albicans* load in the B + I0.1% group was due to the disorganization of the biofilm caused by the mechanical action of brushing. A previous study showed that protocols with an association between brushing and immersion in NaOCl 0.1% concentration showed relevant clinical performance in biofilm control [18]. This difference between studies may have occurred due to the surface roughness of the specimens or dentures analyzed, given that the initial adherence of microorganisms to the surface of a substrate is crucial for its pathogenicity [31].

The protocol that began with immersion in NaOCl at 0.1% before brushing showed greater microbial growth of *C. albicans*. Thus, a prior disorganization of the biofilm, provided by brushing, is necessary for the immersion solution to reach its deeper portions [38]. The brush bristle tips are capable of disorganizing and destabilizing the biofilm’s superficial layers, facilitating exposure of irregularities and pores in the PMMA. They also contribute to a hydrodynamic shear force of fluid flow during brushing, resulting in reductions in fungal load [39]. The choice of using agents like neutral soap facilitates biofilm penetration through the surfactant property of its components, such as sodium lauryl sulfate [17]. The antimicrobial effectiveness of the protocols is linked to their ability to reach microorganisms that adhere to 0.2 μm porosities [40].

Regarding the concentration of NaOCl, the American Prosthodontic Association recommends using 0.5% as a disinfectant solution during a 10 min immersion, coupled with mechanical cleaning using soft brushes [8]. However, previous studies have shown that exposure to NaOCl can lead to discoloration depending on concentration and immersion time, along with an increase in the surface roughness of PMMA [12,17,20,31,41]. The NaOCl at 1% increased the surface roughness of PMMA and caused intermediate color changes [42]. Therefore, the use of sodium hypochlorite at lower concentrations (0.25% and 0.1%) is relevant due to the greater control of potential adverse effects caused by higher concentrations [40]. Arruda et al. [18] determined that a protocol combining mechanical methods with immersion in NaOCl at 0.1% and 0.2%, for 20 min, reduced biofilm compared to the baseline without alterations in color, surface roughness, or flexural strength. In cases of immersion in 0.5%, color changes were observed. Arbeláeza et al. [41], through a viability assay (Alamar Blue), identified that long-term chemical disinfection methods were capable of reducing the metabolic activity of *Candida* fungal cells in multispecies biofilm, despite not reducing colony density (CFU/mL). Their mode of action involves direct interaction with the biofilm’s organic matrix, causing the dissolution of polymeric structures.

In the presence of removable dentures, *C. albicans* can adapt and adhere to both the epithelial surface of the host and the prosthetic base [43]. Thus, its colonization capacity is favored on PMMA [31], which provides, through its rough interface, a suitable site for colonization, especially for mature biofilm development [44]. Therefore, for patients with removable dentures, it is recommended not to wear them during the night due to the potential decrease in the protective effect of saliva, the cleaning action of the tongue, and good mucosal oxygenation [9]. As can be seen, several factors can make it easier for *Candida* strains to colonize dentures, such as poor hygiene, reduced salivary flow, and poor care of dentures [45]. The implementation of a protocol combining disinfection methods and final exposure to the intraoral temperature for 8 h (overnight) aimed to simulate how patients would carry out their cleaning routine and return with the dentures to the oral cavity. Alternately, a systematic review indicated that overnight dry storage is an important option for reducing *C. albicans* colonization, with insignificant alterations to the dimensions of dentures [46].

The B + I + T group achieved reductions of 1.49 log and 1.71 log for immersion concentrations of 0.1% and 0.2%, respectively. It can be inferred that the overnight exposure to the intraoral temperature may have allowed the recolonization of specimens by cells that remained viable after disinfection. The most efficient protocol obtained in this study (B + I0.25%) did not reduce to zero the *Candida* count. O’Donnell et al. [13] demonstrated that, even after denture disinfection, there is still the presence of fungal cells capable of recolonizing the site. These cells impregnated in regions of porosities are more challenging to penetrate due to the protective effects of the biofilm they are enclosed in (efflux pump and extracellular glucans). In the literature, it is reported that *C. albicans* can remain viable by impregnation in irregularities up to 621 μm after 21 days. These cells recognize nutrients present in their substrate and, through a slow process, can recover and emerge again [47]. This finding agrees with previous studies [48,49], recommending that hygiene protocols be performed before storage and preferably dry storage.

The reversal of the sequence of methods in the “overnight” exposure to intraoral temperature protocol was not performed in this study. Given the superior satisfactory results of the B + I protocol in both concentrations compared to the I + B protocol, coupled with the results obtained, adding an I + B + T group was not justified. The water immersion during the denture removal period contributes to controlling the residual release of NaOCl solutions. Allergic reactions to sodium hypochlorite and its irritant action must be considered [50] because it can influence the inflammation level of the palate and alveolar ridge mucosa. Finally, the limitation of this in vitro study was the use of a monospecies biofilm. Future studies examining the symbiotic relationship of *C. albicans* with other microorganisms colonizing the prosthetic surface are necessary. Future studies are also required to relate the monospecific biofilm of *C. albicans* in samples previously submerged in saliva, considering the formation of an acquired film on the structures.

## 5. Conclusions

The most effective intervention sequence started with brushing followed by immersion in NaOCl, with the B + I0.25% group performing the lowest *C. albicans* count. Isolated immersion in NaOCl 0.1% and 0.25% showed the lowest antimicrobial effectiveness. Brushing played a crucial role in reducing CFU/mL of *C. albicans*.

The NaOCl at 0.1%, when used before the brushing method, shows reduced efficiency of the associated protocol (I0.1% + B group). Only NaOCl at 0.25% maintains the efficacy independent of the method sequence.

The intraoral temperature facilitated the recolonization of *C. albicans* after exposure to the disinfection protocols. There was an increase in the average CFU/mL count in both NaOCl concentrations caused by the intraoral temperature.

## Figures and Tables

**Table 1:** Comparison of protocols according to concentration (in %), showing mean and standard deviation values of log_10_ x colony-forming units per milliliter [log_10_(CFU/mL)] for each group.

Groups	Concentrations				*t*-Test	*p*-Value
	0.1%		0.25%			
	Mean	SD	Mean	SD		
I0.1% vs. I0.25%	5.29	0.38	4.97	1.15	<0.05	0.144
B + I0.1% vs. B + I0.25%	3.34	0.42	2.85	0.54	2.569	0.017
I0.1% + B vs. I0.25% + B	3.90	0.31	3.53	0.31	3.000	0.006
B + I0.1% + T vs. B + I0.25% + T	4.47	0.84	4.31	0.95	<0.05	0.555

Legend: *t*-test for independent samples. (I) Immersion, (B) brushing, and (T) intraoral temperature.

## Data Availability

The original contributions presented in the study are included in the article, and further inquiries can be directed to the corresponding author.

## Supplementary Materials

The following supporting information can be downloaded at: https://www.mdpi.com/article/10.3390/polym17010008/s1, Table S1: Descriptive statistics of CFU/mL based on disinfection protocols and NaOCl concentrations; Table S2: Comparison of protocols based on concentration (%) using Tukey’s multiple comparisons test.
