# Supplementary material for: Disinfection Strategies for Poly(methyl methacrylate): Method Sequence, Solution Concentration, and Intraoral Temperature on Antimicrobial Activity
Source: Polymers (Basel). 2024 Dec 24;17(1):8. doi: 10.3390/polym17010008 (PMC11722877; doi:10.3390/polym17010008)
Supplement: Supplementary file 1 [file polymers-17-00008-s001.zip › polymers-3033469-supplementary.pdf]

Supplementary Table S1. Descriptive statistics of CFU/mL based on disinfection protocols and NaOCl concentrations

**Groups**	**C**	**I0.1%**	**I0.25%**	**B**	**B+I0.1%**	**B+I0.25%**	**I0.1%+B**	**I0.25%+B**	**B+I0.1%+T**	**B+I0.25%+T**
**Number of values**	13	13	13	13	13	13	13	13	13	13
**Minimum**	5,740	4,780	3,510	2,680	2,720	1,910	3,480	2,810	3,530	3,300
**Maximum**	6,590	5,880	7,080	4,040	3,900	3,610	4,630	4,270	6,520	6,930
**Range**	0,8500	1,100	3,570	1,360	1,180	1,700	1,150	1,460	2,990	3,630
**Mean**	6,102	5,285	4,973	3,498	3,339	2,854	3,895	3,527	4,468	4,312
**Std. Deviation**	0,2682	0,3766	1,147	0,3805	0,4158	0,5396	0,3092	0,3170	0,8428	0,9470
**Std. Error of Mean**	0,07439	0,1044	0,3181	0,1055	0,1153	0,1496	0,08574	0,08793	0,2337	0,2627

Legend: Comparison of the number of colony-forming units per milliliter (CFU/mL) for various disinfection protocols involving different concentrations of sodium hypochlorite (NaOCl) and combinations of brushing and immersion. The table presents the minimum, maximum, range, mean, standard deviation, and standard error of the mean for each group. (C: Control, I: Immersion, B: Brushing, T: Intraoral temperature).

Supplementary Table S2. Comparison of protocols based on concentration (%) using Tukey's multiple comparisons test.

Tukey's multiple comparisons test	Mean Diff,	SE Diff,	Summary	Adjusted P Value	
C vs. I0.1%	0,8162	0,1438	**	0,0026	A-B
C vs. I0.25%	1,128	0,3284	ns	0,0912	A-C
C vs. B	2,604	0,101	****	<0,0001	A-D
C vs. B+I0.1%	2,762	0,1481	****	<0,0001	A-E
C vs. B+I0.25%	3,248	0,153	****	<0,0001	A-F
C vs. I0.1%+B	2,206	0,1343	****	<0,0001	A-G
C vs. I0.25%+B	2,575	0,08704	****	<0,0001	A-H
C vs. B+I0.1%+T	1,634	0,2516	***	0,0008	A-I
C vs. B+I0.25%+T	1,79	0,3023	**	0,0018	A-J
I0.1% vs. I0.25%	0,3123	0,2983	ns	0,9819	B-C
I0.1% vs. B	1,788	0,1775	****	<0,0001	B-D
I0.1% vs. B+I0.1%	1,946	0,1308	****	<0,0001	B-E
I0.1% vs. B+I0.25%	2,432	0,1762	****	<0,0001	B-F
I0.1% vs. I0.1%+B	1,39	0,1588	****	<0,0001	B-G
I0.1% vs. I0.25%+B	1,758	0,1625	****	<0,0001	B-H
I0.1% vs. B+I0.1%+T	0,8177	0,2296	ns	0,0748	B-I
I0.1% vs. B+I0.25%+T	0,9738	0,2489	*	0,0428	B-J

I0.25% vs. B	1,475	0,3321	*	0,0182	C-D
I0.25% vs. B+I0.1%	1,634	0,3514	*	0,0131	C-E
I0.25% vs. B+I0.25%	2,119	0,3673	**	0,0023	C-F
I0.25% vs. I0.1%+B	1,078	0,3461	ns	0,1497	C-G
I0.25% vs. I0.25%+B	1,446	0,383	ns	0,0532	C-H
I0.25% vs. B+I0.1%+T	0,5054	0,2632	ns	0,659	C-I
I0.25% vs. B+I0.25%+T	0,6615	0,3666	ns	0,7229	C-J
B vs. B+I0.1%	0,1585	0,178	ns	0,9938	D-E
B vs. B+I0.25%	0,6438	0,1783	ns	0,0691	D-F
B vs. I0.1%+B	-0,3977	0,1362	ns	0,199	D-G
B vs. I0.25%+B	-0,02923	0,1282	ns	>0,9999	D-H
B vs. B+I0.1%+T	-0,97	0,2634	ns	0,0617	D-I
B vs. B+I0.25%+T	-0,8138	0,3447	ns	0,4188	D-J
B+I0.1% vs. B+I0.25%	0,4854	0,1919	ns	0,3404	E-F
B+I0.1% vs. I0.1%+B	-0,5562	0,1389	*	0,0369	E-G
B+I0.1% vs. I0.25%+B	-0,1877	0,16	ns	0,9637	E-H
B+I0.1% vs. B+I0.1%+T	-1,128	0,2454	*	0,0142	E-I
B+I0.1% vs. B+I0.25%+T	-0,9723	0,3099	ns	0,1445	E-J
B+I0.25% vs. I0.1%+B	-1,042	0,1763	**	0,0019	F-G
B+I0.25% vs. I0.25%+B	-0,6731	0,1607	*	0,0274	F-H
B+I0.25% vs. B+I0.1%+T	-1,614	0,3225	**	0,0074	F-I
B+I0.25% vs. B+I0.25%+T	-1,458	0,3086	*	0,0116	F-J
I0.1%+B vs. I0.25%+B	0,3685	0,1166	ns	0,1395	G-H
I0.1%+B vs. B+I0.1%+T	-0,5723	0,2692	ns	0,543	G-I
I0.1%+B vs. B+I0.25%+T	-0,4162	0,2732	ns	0,8595	G-J
I0.25%+B vs. B+I0.1%+T	-0,9408	0,2962	ns	0,1362	H-I
I0.25%+B vs. B+I0.25%+T	-0,7846	0,2955	ns	0,2885	H-J
B+I0.1%+T vs. B+I0.25%+T	0,1562	0,3456	ns	>0,9999	I-J

Legend: ANOVA followed by Tukey's multiple comparisons test was conducted to compare the protocols according to their concentration (%). The table presents the mean differences, standard errors (SE), and adjusted P values for Log10 Colony-Forming Units per milliliter [log10(CFU/mL)] across all groups. Significant differences are highlighted based on the adjusted P values. (I) Immersion, (B) Brushing, and (T) Intraoral temperature.
